# Hemangioma in the Third Interdigital Space of the Hand Extending to the Fourth Finger: Surgical Management of a Rare Location

**DOI:** 10.7759/cureus.111731

**Published:** 2026-06-29

**Authors:** Abdelmounaim Daoudi, Sadougui Med, Walid Bouziane, Abdelkrim Daoudi

**Affiliations:** 1 Orthopedics and Traumatology, Centre Hospitalier Universitaire (CHU) Mohammed VI University Hospital, Oujda, MAR

**Keywords:** case report, hand, hemangioma, surgery, vascular tumor

## Abstract

Vascular tumors are uncommon benign soft tissue lesions of the hand and rarely occur within the interdigital web spaces. We report the case of a 57-year-old man who presented with a progressively enlarging painful mass in the third interdigital space of the left hand. Clinical examination revealed a soft, slightly compressible lesion measuring approximately 3 cm, associated with discomfort during grasping activities. Magnetic resonance imaging demonstrated a well-defined, highly vascularized mass extending toward the fourth finger, showing intense contrast enhancement without evidence of bone or tendon invasion. Complete surgical excision was performed through a palmar approach using meticulous dissection to preserve the adjacent neurovascular structures while controlling the feeding vessels. Histopathological analysis confirmed the diagnosis of a cavernous hemangioma with clear surgical margins. The postoperative course was uneventful, with complete recovery of hand function and no evidence of recurrence at six months of follow-up. This case highlights the diagnostic value of magnetic resonance imaging in the evaluation of vascular tumors of the hand and emphasizes the importance of careful surgical planning in anatomically complex regions. Complete excision remains the treatment of choice, providing definitive diagnosis, symptom relief, and excellent functional outcomes. Reporting such rare localizations contributes to improved recognition and management of hemangioma arising within the interdigital spaces of the hand.

## Introduction

Vascular tumors are a heterogeneous group of benign and malignant lesions that originate from blood vessels or vascular structures. Hemangioma is a relatively common tumor, but it remains rare in the hand due to the limited volume of soft tissue and the complex anatomical arrangement of tendons, nerves, and blood vessels [1,2]. Among these unusual locations, involvement of the third interdigital space is exceptionally rare [3,4].

Clinical manifestations are often nonspecific and may include pain, swelling, tenderness on palpation, or functional discomfort during hand movements [5,6]. Imaging techniques such as ultrasound and magnetic resonance imaging (MRI) are useful for preoperative evaluation; however, the definitive diagnosis relies on histopathological examination [7].

We present a rare case of a hemangioma located in the third interdigital space of the hand, which was treated surgically with favorable clinical and functional outcomes.

## Case presentation

A 57-year-old right-handed patient, a manual laborer, presented with swelling that had developed gradually in the third interdigital space of his left hand and had been present for two years. He reported intermittent pain in the fourth metacarpal, which worsened during grasping movements and manual labor, thereby impairing his quality of life. Furthermore, the patient reported no history of trauma, infection, or systemic disease.

Clinical examination revealed a soft, slightly compressible mass, approximately 3 cm in diameter, located in the third interdigital space extending to the fourth metacarpal. The lesion was slightly tender on palpation, with no skin discoloration or signs of inflammation. Finger mobility was preserved, although discomfort was noted during vigorous grasping. The neurovascular examination of the hand was normal.

Standard X-rays showed no bony abnormalities. MRI revealed a mass in the third interdigital space on the palmar aspect of the left hand extending to the proximal phalanx of the fourth metacarpal. This mass is fairly well-defined with regular, semi-circular borders, in close contact with the extensor tendon of the fourth finger and the cortical bone, without invading either. It appears hyper-intense and heterogeneous on T2-weighted images, iso-intense on T1-weighted images, and iso-intensive on T1 factory acceptance testing (FAT) and site acceptance testing (SAT), shows hyper-intensity on short tau inversion recovery (STIR) and diffusion sequences, and enhances intensely and heterogeneously after injection, measuring 3.2×2.2 cm (Figures 1-2).

**Figure 1:**
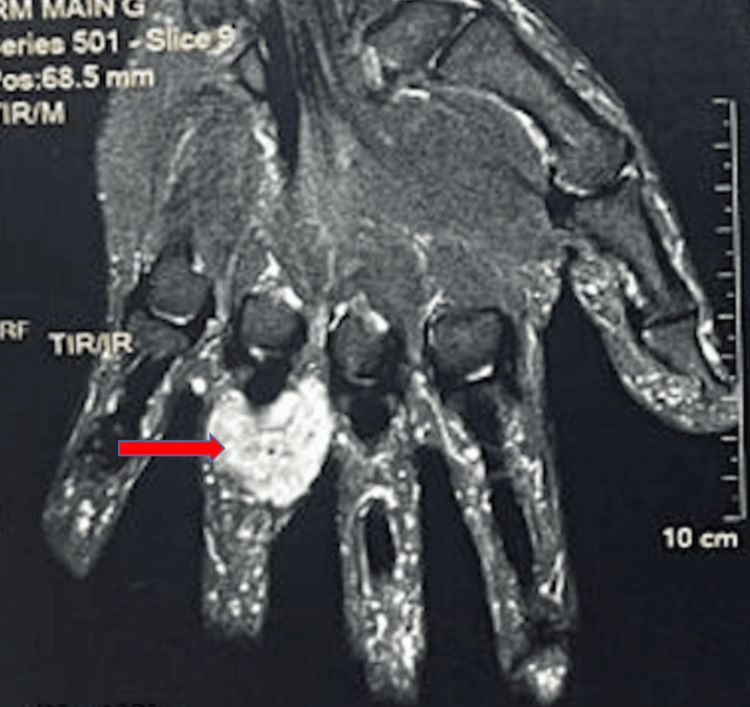
Coronal TIRM (STIR) MR image demonstrating the hyperintense vascular mass (red arrow) within the third web space, without evidence of osseous extension TIRM: turbo inversion recovery magnitude; STIR: short tau inversion recovery; MR: magnetic resonance

**Figure 2:**
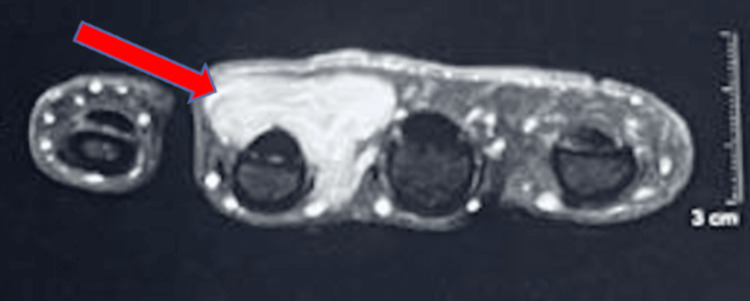
Axial T2-weighted fat-suppressed MR image showing a well-defined hyperintense vascular lesion (red arrow) in the third web space of the left hand MR: magnetic resonance

The patient underwent surgery under general anesthesia with a tourniquet applied to the upper limb. A palmar zigzag incision centered on the third interdigital space and extending toward the proximal part of the fourth metacarpal bone was made to optimize surgical exposure while minimizing postoperative scar contracture.

Meticulous dissection was performed using a magnifying loupe. The mass was encapsulated, highly vascularized, and tightly adherent to the lateral neurovascular bundle of the fourth finger. It encompassed the tendon while respecting its sheath and the cortical bone (Figure 3).

**Figure 3:**
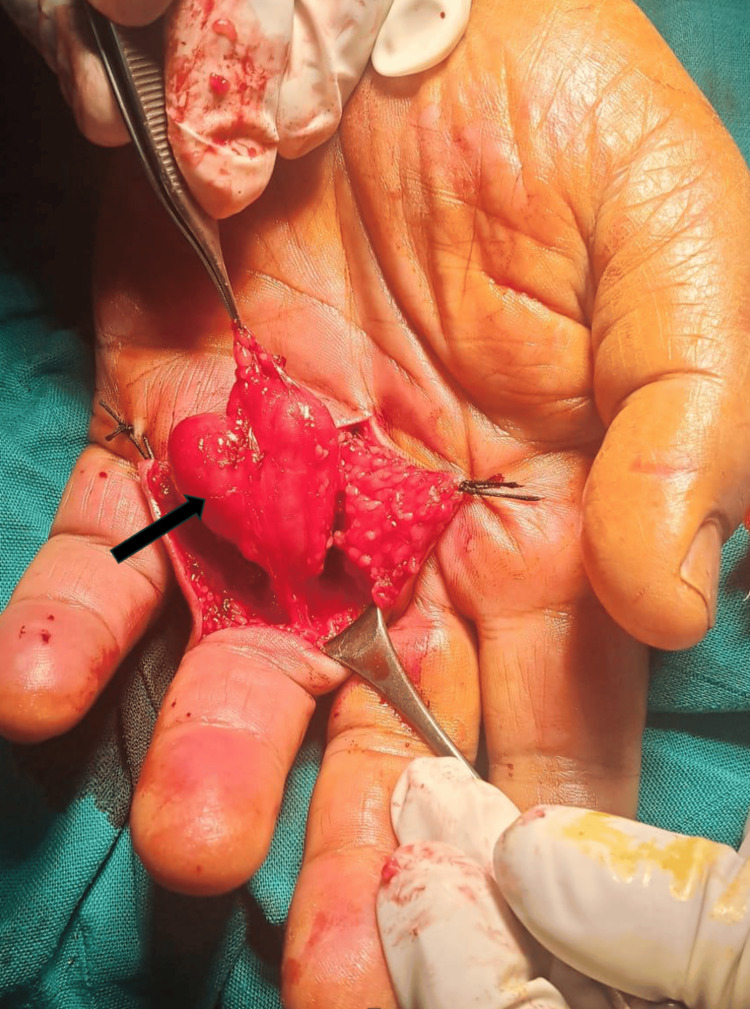
Intraoperative photograph demonstrating the meticulous dissection of a well-defined vascular tumor located within the third interdigital web space. The lesion (black arrow) is isolated from the adjacent digital neurovascular bundles before en bloc excision

The feeding vessels originating from the lateral interdigital artery of the fourth finger were identified and ligated. Subsequent dissection allowed for complete en bloc excision while preserving the surrounding arteries and nerves (Figure 4).

**Figure 4:**
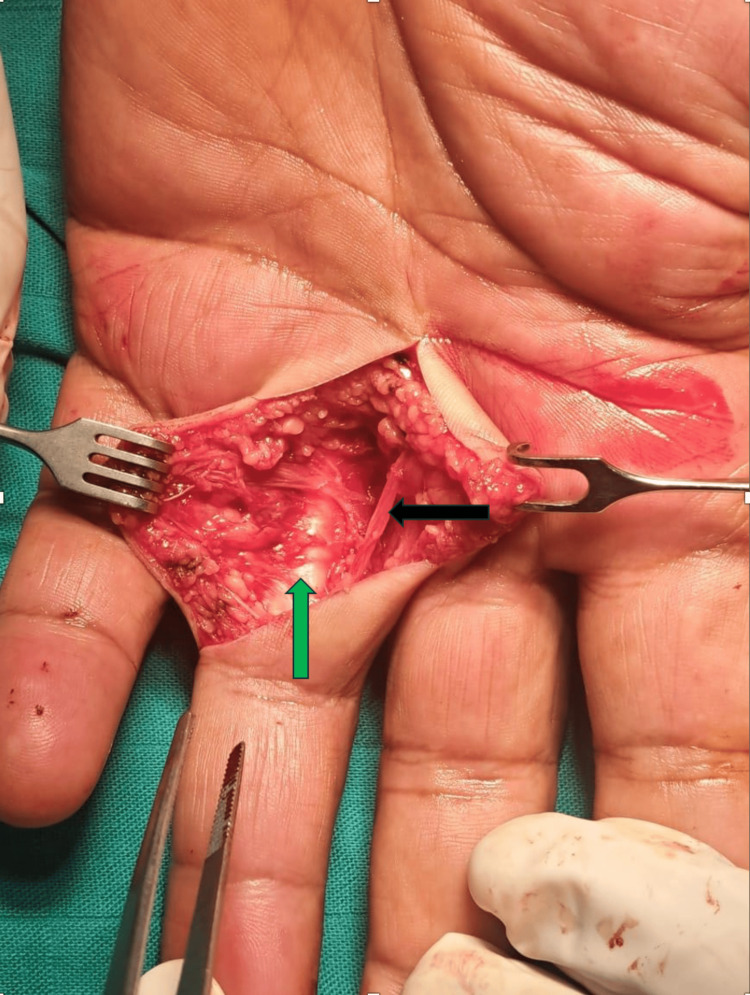
Final appearance after resection of the mass: the green arrow points to the flexor tendon within its sheath, and the black arrow points to the lateral interdigital nerve, which was preserved

Hemostasis was achieved after releasing the tourniquet, and the wound was closed without tension. No skin graft or flap reconstruction was necessary. Histopathological examination revealed the presence of hemangioma with no signs of atypia and clear resection margins (Figure 5).

**Figure 5:**
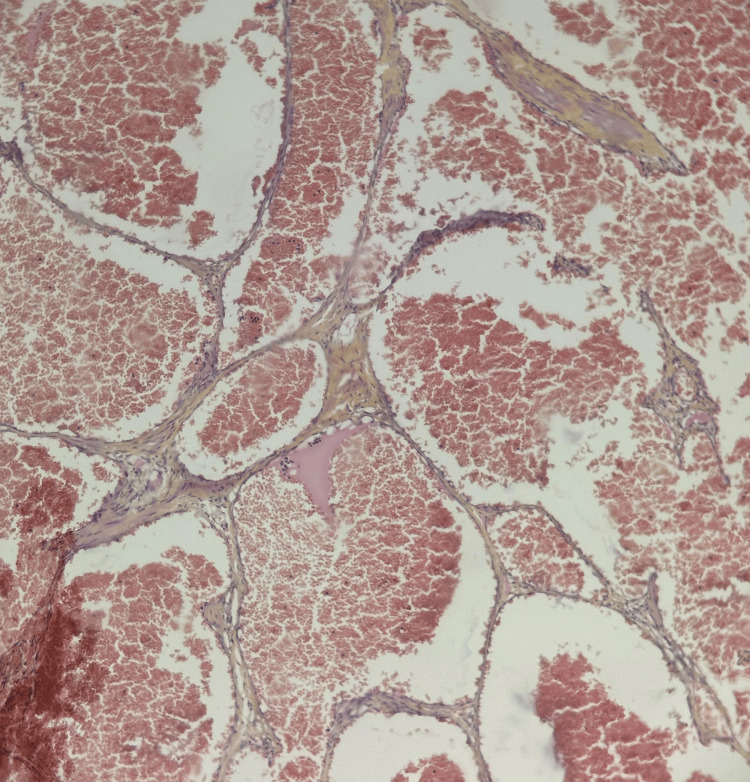
Histopathological examination of the excised lesion stained with Masson's trichrome (original magnification ×40). The lesion consisted of multiple large, blood-filled dilated vascular channels separated by fibrous connective tissue septa and lined by a single layer of flattened endothelial cells without cytological atypia or mitotic activity. No evidence of thrombosis, necrosis, or malignancy was observed. These histopathological findings are compatible with a cavernous-type venous vascular lesion (historically referred to as cavernous hemangioma). Immunohistochemical staining was not performed

The postoperative period proceeded without complications. Early mobilization was initiated on the second day. Sutures were removed at 15 days, with full range of motion of the hand preserved. After three weeks, the patient returned to work as usual. No recurrence was reported after six months of follow-up.

## Discussion

Vascular tumors of the hand are uncommon lesions that account for a small proportion of benign soft tissue tumors encountered in hand surgery. Although vascular anomalies represent a heterogeneous group of lesions, true vascular tumors such as hemangiomas, angioleiomyomas, and spindle cell hemangiomas remain relatively rare in the hand due to the limited soft tissue envelope and the highly specialized anatomical structures present in this region [1,2].

Localization within the interdigital web spaces is particularly unusual. Most vascular tumors reported in the literature occur in the digits, palm, or thenar and hypothenar regions [1,2]. The rarity of web space involvement may be explained by the narrow anatomical compartment containing digital nerves, arteries, lumbrical muscles, and fibrous septa, leaving little potential space for tumor development. In recent reports, lesions affecting the first web space have been described, but even in these series, vascular tumors represent only a minority among masses encountered in this anatomical area [3,4].

Clinically, hemangiomas of the hand usually present as slowly enlarging masses associated with pain, tenderness, or hypersensitivity [5]. These symptoms are often related to the compression of adjacent neurovascular structures or intralesional thrombosis. Because the hand contains numerous anatomical structures within a confined space, even small lesions can produce significant functional symptoms. Patients may therefore present with pain, paresthesia, or mechanical discomfort during hand motion [5,8].

Several reports have emphasized that hemangiomas can mimic more common hand lesions such as ganglion cysts, giant cell tumor of the tendon sheath, epidermoid cysts, or glomus tumors [6,9]. This diagnostic difficulty is compounded by the fact that hemangioma tumors may present without obvious vascular signs such as pulsatility or discoloration. Consequently, imaging studies play an essential role in the diagnostic work-up of these lesions [7,9].

MRI remains the most reliable imaging modality for evaluating vascular tumors of the hand. Typical MRI features include high signal intensity on T2-weighted sequences and variable enhancement following contrast administration, reflecting the vascular composition of the lesion. MRI also allows the precise assessment of the relationship between the tumor and adjacent structures such as digital nerves and arteries, which is crucial for surgical planning [7].

The differential diagnosis of vascular lesions of the hand is broad and includes both benign and malignant entities. Benign tumors such as hemangioma, angioleiomyoma, and intravascular papillary endothelial hyperplasia must be distinguished from malignant vascular tumors including epithelioid hemangioendothelioma or angiosarcoma [2,6]. Although malignant tumors of vascular origin are extremely rare in the hand, several reports highlight the importance of histological confirmation because imaging findings alone may not be sufficient to establish the diagnosis [6,7].

Angioleiomyoma is an uncommon benign vascular tumor that typically presents as a painful, slow-growing mass in adults. In their case series and review of the literature, Yeung et al. reported favorable outcomes following marginal excision, with no recurrence observed during follow-up [5].

Similarly, case reports of hemangiomas involving the digital neurovascular structures have emphasized the potential for neurological symptoms when lesions develop in close proximity to digital nerves, highlighting the importance of meticulous surgical dissection [8].

The present case is notable for its localization within the third web space, which represents an extremely uncommon anatomical site for vascular tumors. Only isolated case reports describing vascular lesions in interdigital spaces have been documented [3,4]. For instance, Yassin and Mohamed recently described a dual-plane hemangioma involving the first web space extending into the adductor pollicis muscle, highlighting the potential for complex anatomical involvement in this region [3].

Surgical excision remains the preferred treatment for symptomatic hemangiomas of the hand. Conservative management may be considered in asymptomatic lesions; however, surgical intervention is generally indicated when the lesion produces pain, progressive enlargement, or functional impairment [1,2]. Complete excision provides both definitive treatment and histopathological confirmation of the diagnosis.

Surgery in the interdigital space requires particular caution because of the proximity of digital neurovascular bundles. Microsurgical dissection and magnification are often recommended to minimize the risk of iatrogenic injury [1,8]. Previous reports have demonstrated that careful surgical technique allows complete tumor removal while preserving digital nerves, arteries, and tendons, resulting in excellent functional outcomes [1,5].

In addition to surgical excision, several adjunctive treatment modalities have been described for vascular anomalies of the hand, including sclerotherapy, embolization, or laser therapy. However, these techniques are more frequently applied to vascular malformations rather than true vascular tumors [1,2]. For localized hemangiomas, marginal excision remains the most reliable and definitive therapeutic option [5].

Histopathological examination remains the gold standard for diagnosis [2,6]. Depending on the tumor subtype, microscopic findings may include proliferation of endothelial cells forming vascular channels of variable size, smooth muscle components, or thrombosed vascular spaces [2,6]. Histological evaluation is also essential to rule out malignant vascular neoplasms, which require more aggressive management strategies [2,6].

The prognosis following complete excision of benign vascular tumors of the hand, such as hemangiomas, is generally excellent. Recurrence is uncommon but may occur in cases of incomplete resection or infiltrative growth patterns. Long-term follow-up is therefore recommended, particularly for lesions located in anatomically complex regions such as the web spaces where complete resection may be technically challenging [1,5].

## Conclusions

This case adds to the still-limited literature describing hemangiomas occurring in the interdigital spaces of the hand. It is important to report these rare locations in order to improve clinical recognition and guide appropriate management. Knowledge of these uncommon tumors is of particular importance to hand surgeons, as early diagnosis and meticulous, well-managed surgical treatment can prevent functional deficits and ensure favorable outcomes.
